# A study on the dynamics of temporary HIV treatment to assess the controversial outcomes of clinical trials: An *in-silico* approach

**DOI:** 10.1371/journal.pone.0200892

**Published:** 2018-07-18

**Authors:** Emiliano Mancini, Rick Quax, Andrea De Luca, Sarah Fidler, Wolfgang Stohr, Peter M. A. Sloot

**Affiliations:** 1 Institute for Advanced Study, University of Amsterdam, GC Amsterdam, The Netherlands; 2 Azienda Ospedaliera Universitaria, UOC Malattie Infettive Universitarie, Siena, Italy; 3 University College London Medical School, London, London, United Kingdom; 4 Nanyang Technological University, Singapore, Singapore; 5 ITMO University, St. Petersburg, Russian Federation; University of Nebraska Medical Center, UNITED STATES

## Abstract

It is still unclear under which conditions temporary combined antiretroviral therapy (cART) results in a prolonged remission after interruption. Clinical trials have contradicting reposts about the effect of cART during primary HIV infection on the disease progression. Here we propose that the apparent contradiction is due the presence of a window of opportunity for cART treatment observed in the *in silico* studies. We study non-linear correlations in the HIV dynamics over time using information theory. This approach requires a large dataset of CD4+ T lymphocytes and viral load concentrations over time. Since it is unfeasible to collect the required amount of data in clinical trials we use C-ImmSim, a clinically validated *in silico* model of the HIV infection, to simulate the HIV infection and temporary cART in 500 virtual patients for a period of 6 years post infection in time steps of 8 hours. We validate the results of our model with two published clinical trials of temporary cART in acute infection and analyse the impact of cART on the immune response. Our quantitative analysis predicts a “window of opportunity” of about ten months after the acute phase during which a temporary cART has significantly longer-lasting beneficial effects on the immune system as compared to treatment during the chronic phase. This window may help to explain the controversial outcomes of clinical trials that differ by the starting time and duration of the short-term course cART and provides a critical insight to develop appropriate protocols for future clinical trials.

## Introduction

Treatment interruptions are still a controversial topic [1–7] despite the fact that the general opinion agrees on the Test & Treat strategy which recommends continuous treatment as soon as HIV infected patients are identified [8]. The scientific community disagrees on two points: the risks associated to the voluntary interruption of treatment [2] and the need to stop cART as the only true test to evaluate “functional cure” or “virologic remission” for novel strategies exploring “HIV-cure” [3]. Assuming clinical studies are expected to use HIV treatment interruptions to verify the duration of virologic remission and the efficacy of strategies for a cure, understanding the effect of temporary cART is extremely important. In this context the contradictory results of clinical trials pose a problem which needs to be addressed.

A recent study assessing long term outcomes for patients on cART reported that treatment interruptions longer than 3 months have significant negative effects on immune recovery [2]. Other studies report no significant changes in the viral setpoint [9–11] or a worsening of the disease progression [12,13]. On the other hand, several studies in patients with primary HIV infection report benefits of temporary cART such as lowering of the viral setpoint [14, 15] and limitation of viral reservoirs [16, 17]. For instance, a recent clinical trial reports positive effects of temporary cART during the first 6 months post infection [18]. An explanation for these conflicting results remains elusive [4].

*In silico* studies provide a feasible and ethical alternative to investigate different treatment scenarios [19, 20–23] and allow to simulate quantitatively the immune response to the HIV infection dynamics. Our goal is to explain why and under what conditions a temporary antiretroviral treatment might achieve the observed long-term beneficial effects. We do this by studying the long-lived correlations in the system using information theory. To this end we define the Sustained Response Score (SRS). SRS is a measure of the time it takes for the non-linear correlation between two specific dynamic variables, the concentration of CD4+ T helper lymphocytes (CD4+ cells) and the size of the HIV reservoir (Provirus), to disappears from the system. Correlation is a necessary but not sufficient prerequisite for causal effect. Since HIV-1 virus infects primarily CD4+ cells, the causal link between the two variables is proven. Given the causal relation between CD4+ cells and Provirus, SRS indicates the duration of the beneficial effects of a temporary cART administered at a given time post-infection.

A large dataset of the concentration of T helper lymphocytes and viral load over time is necessary to achieve the statistical significance necessary to compute the mutual information and consequently SRS. Data from hospitals and clinical trials is too sparse, providing only up to a few data points per patient. Therefore, we generate an appropriate dataset by simulating the HIV infection in 500 virtual patients for a period of 6 years post infection in time steps of 8 hours. We perform the simulations using a clinically validated and well established Immune System model [19, 20–23]. We evaluate the results obtained from these *in silico* studies against data from two clinical trials [11, 18] which reported discordant beneficial effects.

The predicted window of opportunity for temporary cART treatment provides a novel insight in the HIV infection dynamics. The existence of a period of high (non-linear) correlation between the amount of T helper cells and the size of the viral reservoir has never been observed before. A temporary treatment outside such window of opportunity is predicted to have short term beneficial effects as reflected by a lower level of sustained response score. As a consequence SRS appears to provide an explanation to the contradictory results of existing clinical trials. Additionally, the methodology that led to this discovery is novel, since this is the first application of tools that measure mutual information and information dissipation time on a model of the human immune response to HIV.

## Materials and methods

**Fig 1:**
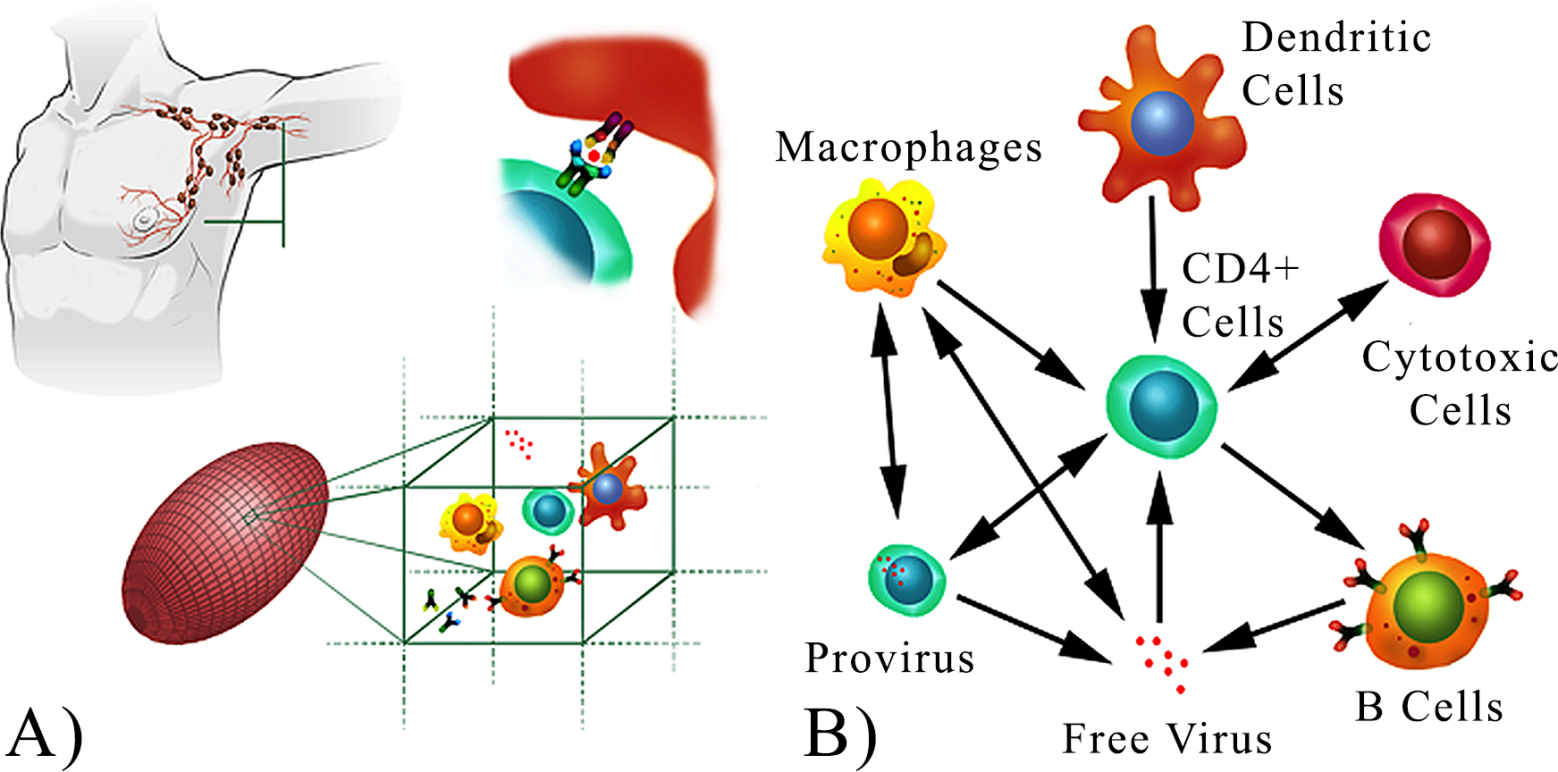
*In silico* model of HIV infection. (A): The *in silico* experiments are performed in a 3-dimensional ellipsoidal lattice. The lattice represents a lymph node with a volume of 4 microliters. Each lattice point represents a microscopic volume in which stochastic interactions occur between the modeled entities. Some of the entities in the model are shown in the sketch: CD4+ T-lymphocytes, B-lymphocytes, antibodies (Ab), macrophages (MA), dendritic cells (DC) and interferon γ.(B): The interactions between the main cellular compartments in the adaptive immune response. This network describes the adaptive immune response to HIV, that in our model is simulated by a three dimensional agent-based model of a lymph node. The virus can be in two forms: free virus (infectious virions) and viral genome copies contained in both silently and actively infected cells (provirus). The complexity of this network does not lie in its topology but rather is hidden in the complex spatio-temporal interactions (arrows) between the nodes.

Key mechanisms of the adaptive human immune response like cross-reactivity, affinity maturation and the complex interplay of the HIV virus with the different subsets of immune cells play a fundamental role in the HIV dynamics. We model the adaptive immune response to HIV infection in each virtual patient by simulating individual immune cells, HIV viral particles, and cytokines, which interact in a volume of four microliters of a lymph node (Fig 1A) according to a specific network of interactions (Fig 1B). For this purpose we use C-ImmSim, a validated agent-based model of HIV infection. The free parameters of this model have been optimized and validated against clinical data over the past 10 years [19–24] ensuring the model is a reliable *in silico* simulation of the HIV infection. We did not further adjust the model parameters for this work. In fact C-ImmSim simulates the underlying dynamics of both virus and adaptive immune response more closely than mathematical models [25–28]. The study of HIV through cellular automata and agent-based models is quite common [19–24, 29, 30] due to the discrete nature of the biological entities involved as well as the complexity of their collective dynamics. The main advantage of C-ImmSim over mathematical models is that it allows to simulate the whole disease progression in all its stages whereas sophisticated mathematical models report issues to reproduce with a good accuracy the infection in all its stages [31] or to reproduce the dynamics with the clinically observed log-normal distribution of time to AIDS [32]. In addition C-ImmSim takes into account HIV mutations and different sub-populations of each cell type, distinguished by their specificity to the HIV virus. C-ImmSim has been used to investigate the differences in early versus deferred treatment and its predictions regarding the role of provirus as a barrier to HIV eradication [21] have been recently confirmed by new observations on the role and size of latent HIV reservoirs [17, 33].

### Virtual clinical data

We randomly vary three parameters to generate 500 different virtual patients: the first two parameters assign unique bit-strings describing the two major histocompatibility complexes (MHC-I and MHC-II) expressed on the membrane of immune cells of a specific virtual patient. The third parameter distributes up to 4096 different MHC-I and MHC-II cell receptors over the population of cells for each patient. The clinical data used for this analysis consists of cell counts per microliter for each of the main cellular entities involved in the adaptive immune response: T helper lymphocytes (CD4+ cells), cytotoxic lymphocytes, macrophages, B cells and dendritic cells. In addition, the data includes viral particle counts per milliliter (viral load) and the amount of virus inside the infected cells per microliter (provirus). The model stores both cellular and viral data for every 8 hours of simulated time from a single lymph node of 4 microliters for each of the 500 different virtual patients. A limitation of the model is that it simulates the immune response to the HIV virus only in a lymph node and not in other types of lymphatic tissues that have different cellular densities. The impact of this limitation has been proven to be non-significant during the validation of the model in previous work. Our model of the HIV response in a lymph node replicates the dynamics of the HIV infection in an infected patient including its viral load, CD4+ cell counts and time to AIDS.

### Sustained response score

The Sustained Response Score (SRS) is designed to measure the characteristic time of decay of the time-delayed correlation of one dynamic (stochastic) variable (*X*) to another (*Y*). These variables are the concentrations over time in the *in-silico* experiments such as CD4+ cell count, macrophages, B cells and dendritic cells. These concentrations are stochastic (i.e., are characterized by a probability distribution) at each time point because of the multiple, randomized initial conditions as well as the stochasticity of the interactions in C-ImmSimm.

The time-delayed correlation between one variable *X(t)* and the future state of another variable *Y(t+Δ)* is quantified here by their mutual information [34]:

$$I\left(X(t),Y(t+\Delta )\right)=\sum_{x}\sum_{y}p(x,y)\frac{p(x,y)}{p(x)p(y)}.$$ (1)

Where *p(x*,*y)* is defined as the joint probability that *X(t) = x* and *Y(t+Δ) = y*; *p(x)* and *p(y)* are the corresponding marginal probabilities. As an example, for *t = 0*, *Δ = 1*, *X* the CD4+ cell concentration and *Y* the macrophages concentration, Eq (1) expresses the correlation between the initial CD4+ concentration and the subsequent macrophages concentration one model time step in the future. Mutual information is a measure of the mutual dependence between two variables and is well-established information-theoretic measure which captures both linear as well as non-linear correlations. It is equal to zero if and only if *X(t)* and *Y(t+Δ)* are independent, i.e., *p(x*,*y) = p(x)p(y)*. In the other extreme it attains its maximal value if and only if *Y(t+Δ)* is always uniquely determined given the value for *X(t)*, or vice versa. For non-linear relationships it is possible that the (linear) Pearson correlation yields a value of zero while the mutual information is no-zero or even maximal [35, 36]. An example would be Y = X^2^ and Y = sin(2π X) in the interval [0,1]. In this case the Pearson correlation between the two functions yields a value of 0 while the mutual information is non-zero.

**Fig 2:**
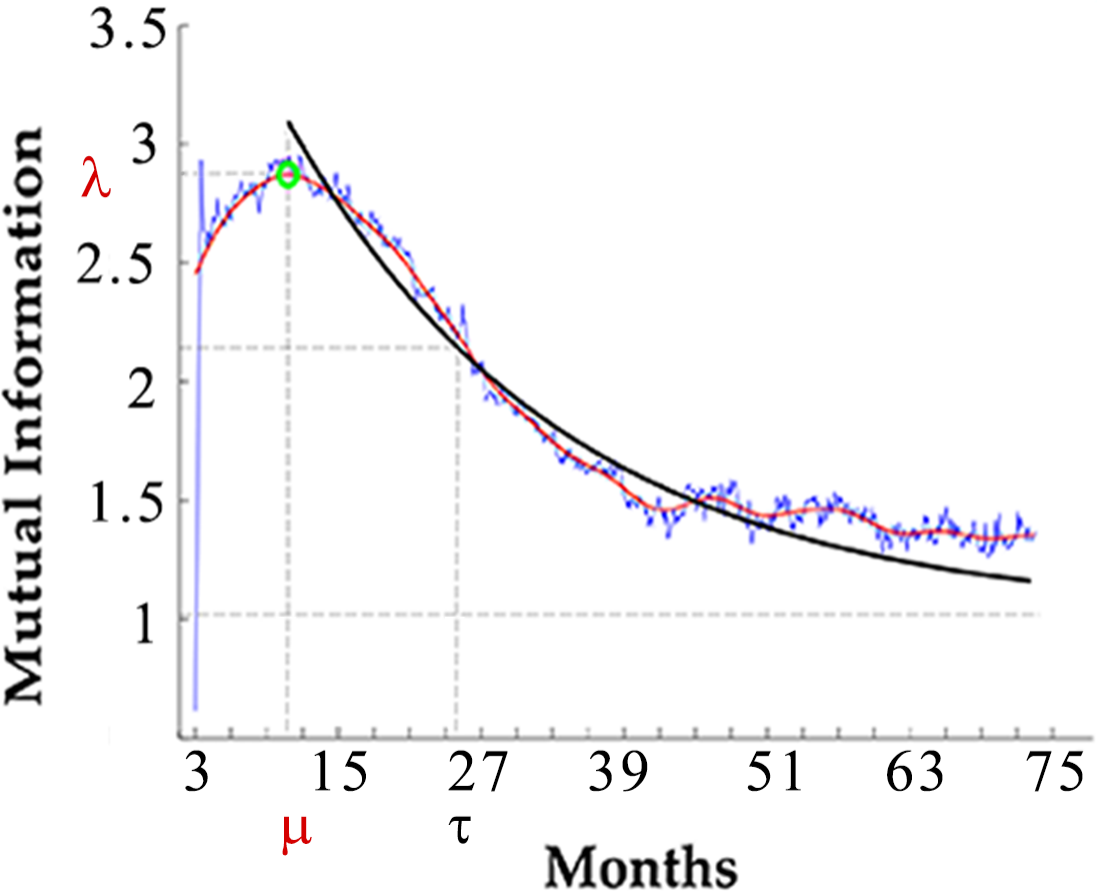
Mutual information at 3 months post infection. Mutual information is a measure of the correlation of one entity over time to changes in one entity at A given time. The maximum correlation between the two entities is the peak *λ*, indicated by the green circle. Its value denotes maximum mutual information between the two entities. Since mutual information decays roughly exponentially after the peak (not necessarily converging to zero), we first determine the time of the peak (μ) and then determine the halftime of the decay (*τ*) from the peak onward. The curve fitting was performed over all the data points after the peak of mutual information. We used an exponential curve fitting to calculate the time at which 50% of the information over the baseline was lost, known as information dissipation time.

We define the Sustained Response Score (SRS), also denoted as *S*_*X*→*Y*_*(t)*, as the characteristic time at which *I*(*X*(*t*),*Y*(*t*+Δ)) decays to a (very small) constant as function of the time step Δ. Calculating SRS involves several steps because we observe that *I*(*X*(*t*),*Y*(*t*+Δ)) typically first grows to a maximum correlation value and then decays roughly exponentially (not necessarily converging to zero) as function of Δ (Fig 2). Therefore, we first determine the time of the peak (μ) and then determine the halftime of the decay (*τ*) from the peak onward also known as information dissipation time [37, 38]. Their sum (*μ* + *τ*) already characterizes the total time it takes for the (non-equilibrium) correlations to disappear. However, it ignores the absolute value of the correlations. Since large correlation values are a necessary prerequisite to large perturbation impacts, we account for this by multiplying *μ* + *τ* with the height of the peak *λ*.

Written mathematically:

$$SRS(t)=S_{X\to Y}\left(t\right)\equiv \lambda \cdot \left(\mu +\tau \right),$$ (2)

where

$$\begin{array}{l} \mu =\arg\;\max_{\Delta }I\left(X(t):Y(t+\Delta )\right),\\ \lambda =I\left(X(t):Y(t+\mu )\right),\\ \tau =\{\Delta \;\mathrm{such}\;\mathrm{that}\;I\left(X(t):Y(t+\Delta )\right)=\frac{1}{2}\cdot \lambda \;\mathrm{and}\;\Delta >\mu \}. \end{array}$$ (3)

In summary, a low SRS means that a (temporary) treatment administered at time *t* necessarily has only a short-lived effect, as there cannot be causation without correlation [39]. On the other hand, a high SRS means that the causal effect of *X* on *Y* potentially lasts for a long period of time. The only way in which a high SRS value between *X* and *Y* could be a ‘false positive’, i.e. the effect turns out to be actually short-lived, is by a confounding third variable *Z* which induces these non-causal correlations through interactions *X* ← *Z* → *Y*. However, assuming the network of interactions in Fig 1, we discount the existence of such a confounder in each case, implying that all high SRS values we find are deemed of causal nature.

## Results

**Fig 3:**
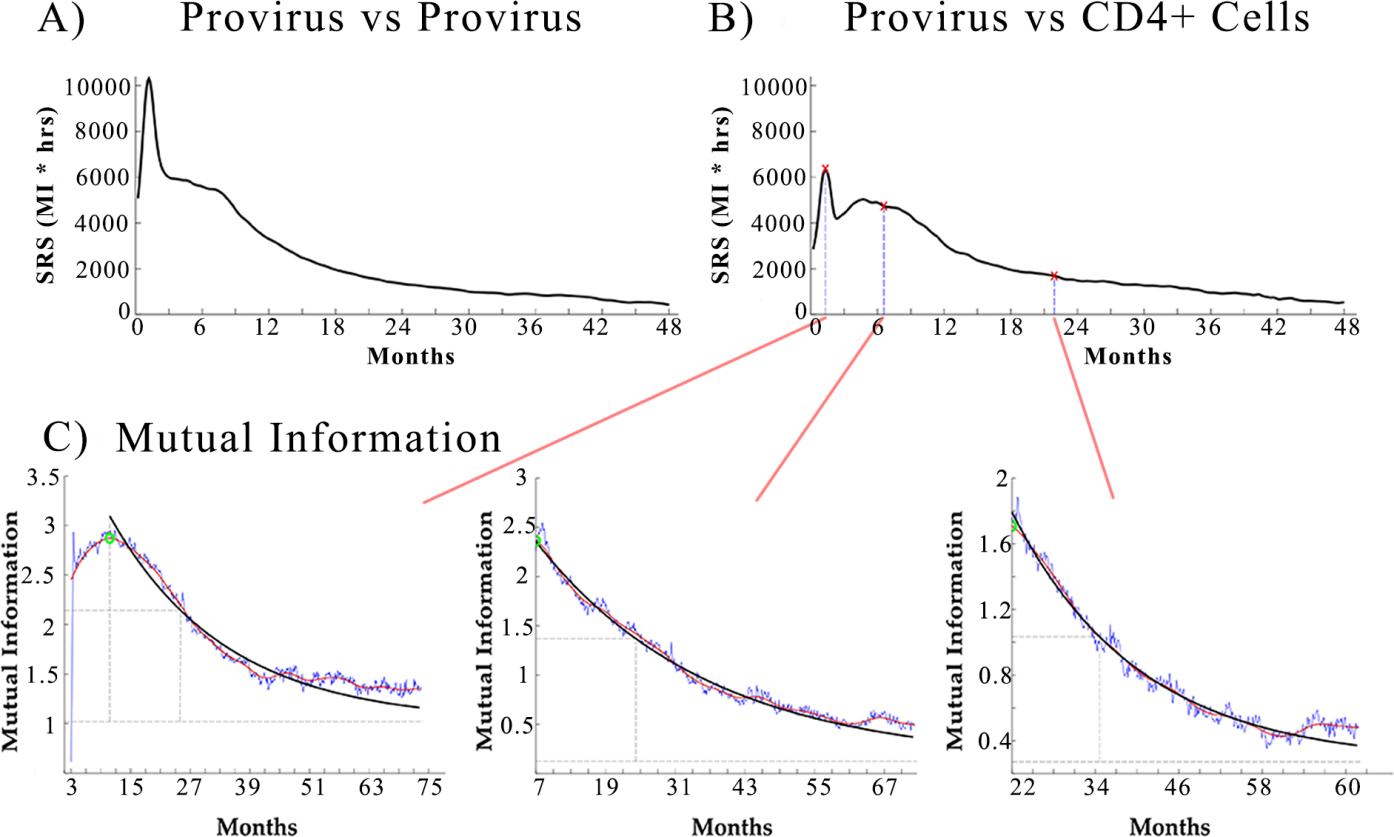
SRS over time of provirus with provirus and CD4+ cells counts. SRS is proportional to the duration of the response of the immune system to a temporary treatment. A high SRS at time t means the beneficial effect of treatment started at time t will last longer. (A) The SRS over time of provirus vs provirus is calculated on the auto-mutual information of the provirus time series. It shows the effect on the level of provirus over time due to a change in provirus at a certain time. (B) The SRS over time of provirus vs CD4+ cells counts shows the effect on the CD4+ cell count due to a change in the provirus. Both panels A and B show a peak in the SRS of the provirus during the acute phase of the HIV infection. After this peak we observe a plateau (until 10 months post infection) with a SRS about 5 times higher that the SRS of the chronic phase on the infection. The plateaus show the increased beneficial effects of cART during the first 10 months post infection. The red markers in panel B represent the SRS calculated from the graphs shown in panel C. (C) (lower set of 3 graphs): Mutual information for provirus at time t versus CD4+ cells over time. Mutual information is a measure of the correlation of the CD4+ cell count over time to changes in the Provirus at various times (90 days, 180 days and 500 days respectively). From a medical perspective it measures the impact on the CD4+ cells recovery if the size of the HIV reservoir is affected by treatment with cART at a given time.

We focus our analysis on the three most important indicators of the HIV infection: CD4+ cells (T helper lymphocytes), provirus (the amount of HIV-1 DNA included in infected cells) and viral load (log_10_ viral RNA copies/ml). The key elements to evaluate the progression of HIV-associated disease in a patient are its CD4+^+^ cell count and the amount of viral genome copies in the infected cells (provirus), since their role in the survivability of HIV-1 infected virtual patients is well characterized [17, 21]. The role of provirus in the HIV infection has been recently reassessed experimentally, indicating that the size of replication-competent HIV reservoirs can be up to 60-fold greater than previously estimated [33]. Early treatment has been reported to affect the size of HIV reservoirs [11] and the viral set point [9, 10], both directly related to the provirus. For these reasons the SRS of the provirus with respect to other indicators (Fig 3A and 3B) is a relevant indicator for the most appropriate timing of a treatment.

Our results confirm that the immune system had the longest sustained response to treatment during the acute phase of the HIV infection, as indicated by a very high SRS during the first two months after infection. In addition to this phase the results indicate a transient phase during which the SRS plateaus are still at a relatively high value. The duration of this phase is roughly ten months. In Fig 3 we show the SRS of provirus with provirus and CD4+ cells counts. Each point of the SRS curve is the product of the mutual information at the peak (green marker in Fig 3C) and the sum of the delay in the mutual information peak plus the information dissipation time. The use of mutual information and the information dissipation time is a new approach to identify non-linear pair-wise correlations in complex systems that has been proven to be better than more conventional correlations [38, 40, 41]. In Fig 3C each graph shows the change in mutual information in the CD4+ cells over time generated by a perturbation of the provirus level at the initial time point (respectively at about 2, 6 and 22 months post infection as indicated by the red markers in Fig 3B).

### CD4+ cells sustained response score

**Fig 4:**
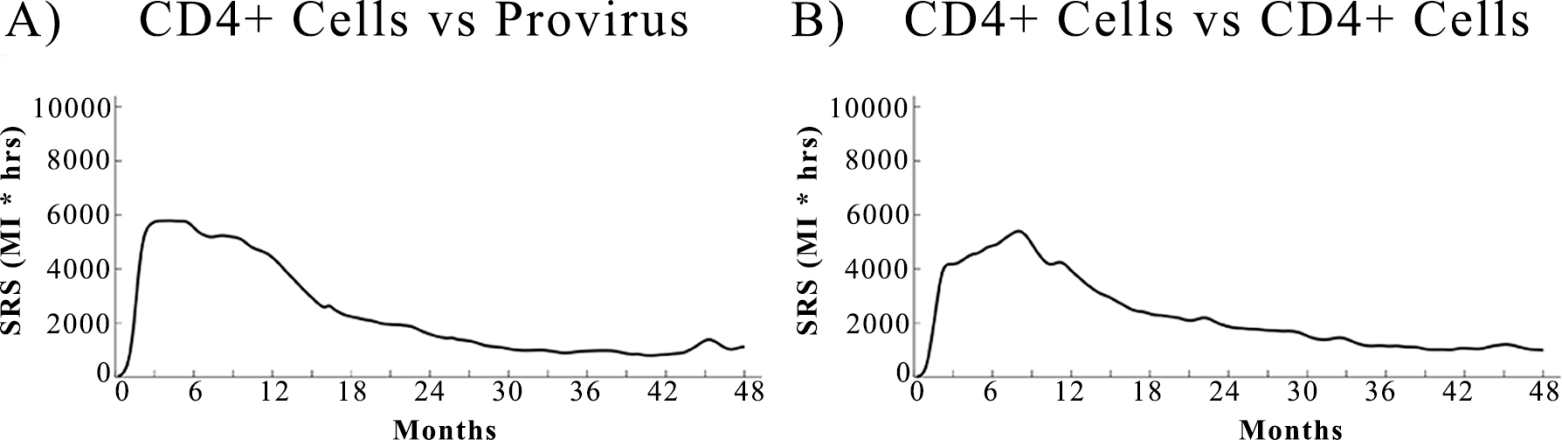
SRS over time of CD4+ cells count with provirus and CD4+ cells counts. SRS is proportional to the duration of the response of the immune system to a temporary treatment. (A) The SRS over time of CD4+ vs provirus shows a plateau in the SRS in the first 12 months post infection. (B) The SRS over time of CD4+ vs CD4+ is calculated on the auto-mutual information of the CD4+ time series. It also shows a plateau in the SRS in the first 12 months post infection. In both panels the SRS of the plateau is about 5 times higher than the SRS relative to the chronic phase of the infection. A high SRS at time t means the beneficial effect of treatment started at time t will last longer. The plateaus in the two panels show the increased beneficial effects of cART during the first 10 months post infection.

The sustained response score for CD4+ cells in the first ten to twelve months post infection, shown in Fig 4A and 4B, is significantly higher than the baseline, where the baseline is defined as the SRS obtained when treatment is initiated during the chronic phase of the infection. For the CD4+ cell count we find that there is only one phase of sustained response due to treatment that lasts up to 12 months post infection. Interestingly, during the acute phase the SRS of CD4+ cells is low. This effect could be due to the fact that CD4+ cells may rapidly undergo apoptosis upon infection during this phase and therefore will not be preserved. On the other hand, the phase of high SRS could mark the period during which a fluctuation in the total amount of T lymphocytes might have a longer lasting, albeit temporary, effect on the balance between the ability of the immune system to respond to the infection and the size of HIV reservoirs established in the system.

### Comparison against two clinical trials

Our predictions are based on the *in silico* infection dynamics. In this section we compare the infection dynamics in virtual patients with that of patients from two clinical trials which shared their data. The PRIMO-SHM clinical trial reported the beneficial effects of a period of 24 and 60 weeks of temporary cART when started within the first 6 months post infection [18]. In this trial 173 patients were randomized. The temporary cART transiently lowered the viral set point and delayed the time at which the CD4+ cell count of treated patients dropped below 350 cells/ml.

The SPARTAC trial also reported beneficial effects associated to a short course treatment for a period of 48 weeks [11] but the entity of such beneficial effects were minor if compared to the ones reported by the PRIMO-SHM.

**Fig 5:**
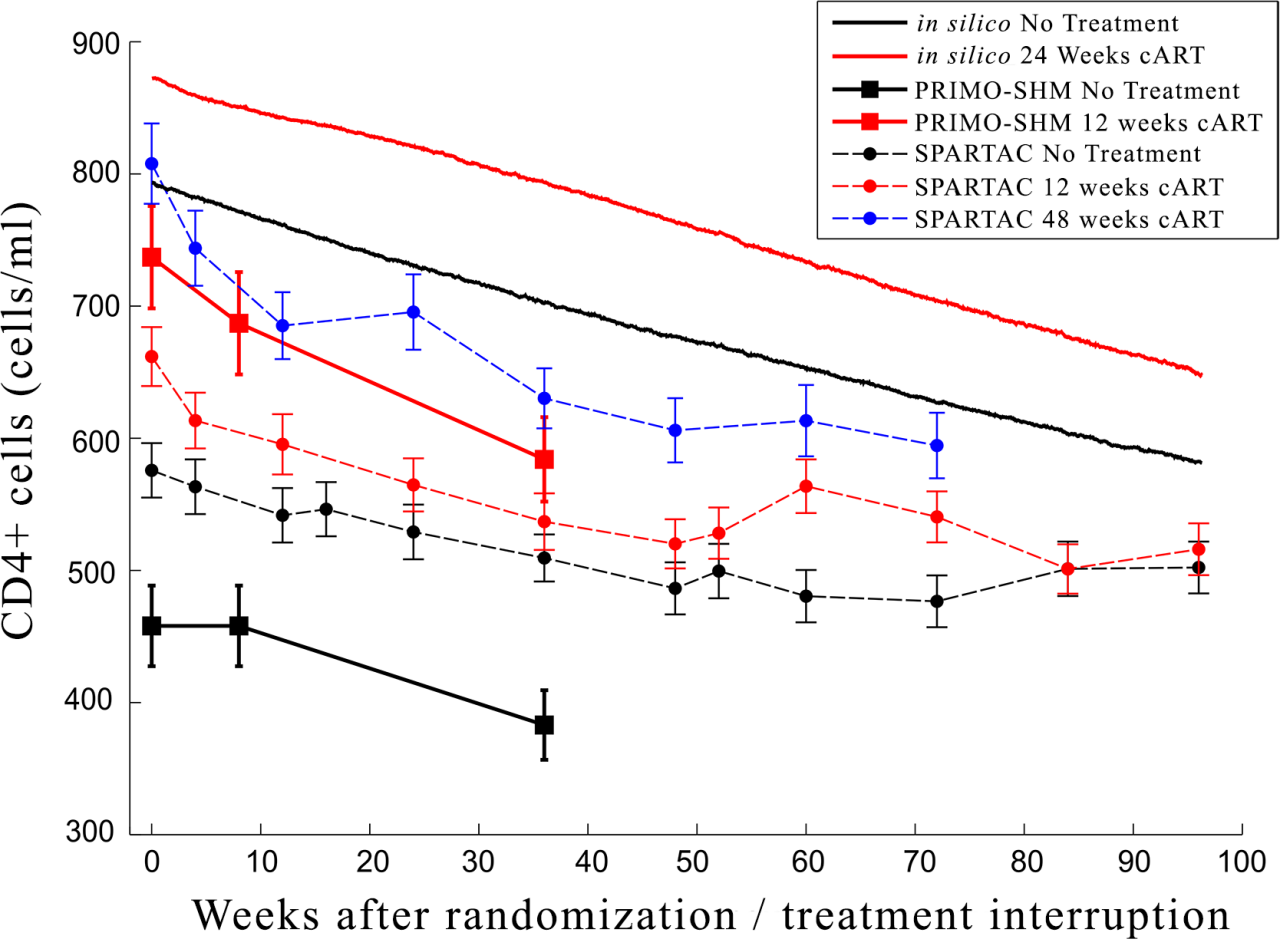
Comparison of the CD4+ cell counts between *in silico* experiments and clinical trials. Comparison of the *in silico* experiments (continuous lines) with clinical data of the PRIMO-SHM study group (circle and square markers) on CD4+ cell count after randomization/treatment interruption in the no treatment and treatment arms. The dynamics are comparable since the curves differ only by a constant factor. In other words, the rate of the decrease in the concentraition of CD4+ cells over time is the same for both virtual patients and patients from the two clinical studies. The difference lies only in the initial amount of CD4+ cells. Clinical trials selected patients with a symptomatic acute infection probably due to a weaker immune response at the time of infection. In the model we were not able to discriminate virtual patients between those with a stronger or weaker immune system at the time of the infection and this explains the difference in the initial number of CD4+ cells.

**Fig 6:**
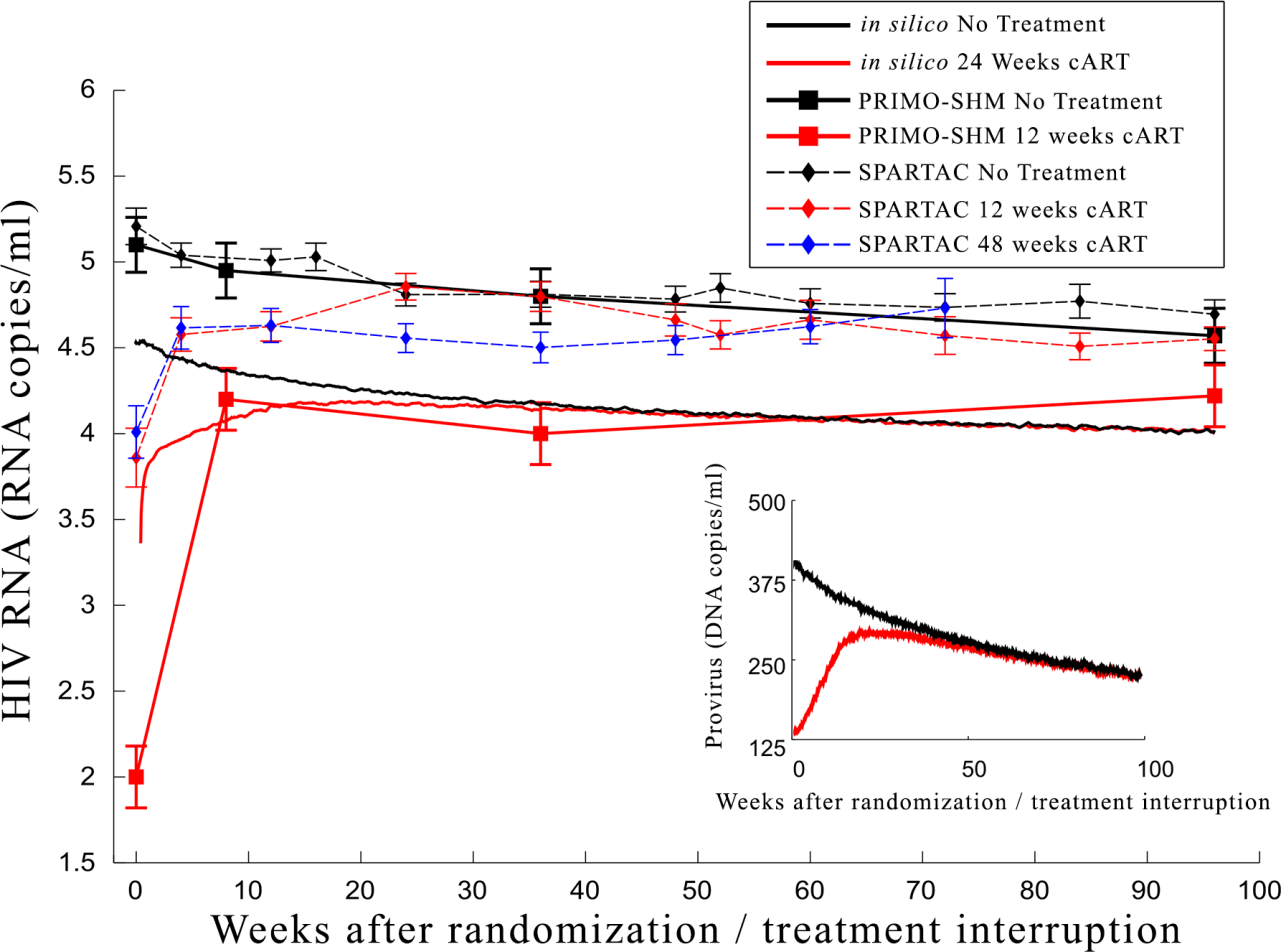
Comparison of the HIV RNA between *in silico* experiments and clinical trials. Comparison of the *in silico* experiments (continuous lines) with clinical data of the PRIMO-SHM study group (circle and square markers) on viral load after randomization/treatment interruption in the no treatment and treatment arms. The dynamics are qualitatively comparable since the curves differ only by a scaling factor. In fact, the *in silico* provirus concentration decreases with a rate similar to the one shown in the clinical trials. The difference between clinical trials and *in silico* simulations in the initial value of provirus of the untreated branch is likely related to the selection of patients with a symptomatic infection during the primary phase in both clinical trials. As previously discussed, that selection could not be performed in the *in silico* simulations. In the inset we show the prediction of the effect of temporary cART on the size of HIV reservoirs after treatment interruption (data not available in the clinical study). In the inset we show the prediction of the effect of temporary cART on the size of HIV reservoirs after treatment interruption (data not available in the clinical study).

We compare the simulation results for 500 virtual patients with the clinical data from PRIMO-SHM and SPARTAC studies (Figs 5 and 6). In absolute terms the simulation results are shifted by a constant factor compared to the PRIMO-SHM clinical data. In other words, the rate of the decrease in the concentration of CD4+ cells over time is the same for both virtual patients and patients from the two clinical studies. The difference lies only in the initial amount of CD4+ cells. PRIMO-SHM clinical trial selected patients with a symptomatic acute infection likely due to a weaker immune response at the time of infection. In this model we were not able to discriminate virtual patients between those with a stronger or weaker immune system at the time of the infection and this explains the difference in the initial number of CD4+ cells. This explanation is supported by the lower CD4+ cell counts observed in the clinical trial in comparison to the simulated ones. A more aggressive infection in the patients selected in the PRIMO-SHM trial might also explain the stronger effect of temporary treatment on the viral set point observed in their study. Comparison with SPARTAC data shows a similar trend. Again *in-silico* data differs by a constant scaling factor, but the difference is smaller since SPARTAC trial included both patients with and without a symptomatic acute phase.

Qualitatively the temporary treatment has a long lasting effect on both CD4+ cell counts and viral load in both *in silico* experiments and *clinical* data. In our experiments the viral load converges to the untreated level after roughly one year, faster than observed in the Primo-SHM study. Conversely the CD4+ cell counts in our experiments show the same dynamics as in the clinical study.

**Fig 7:**
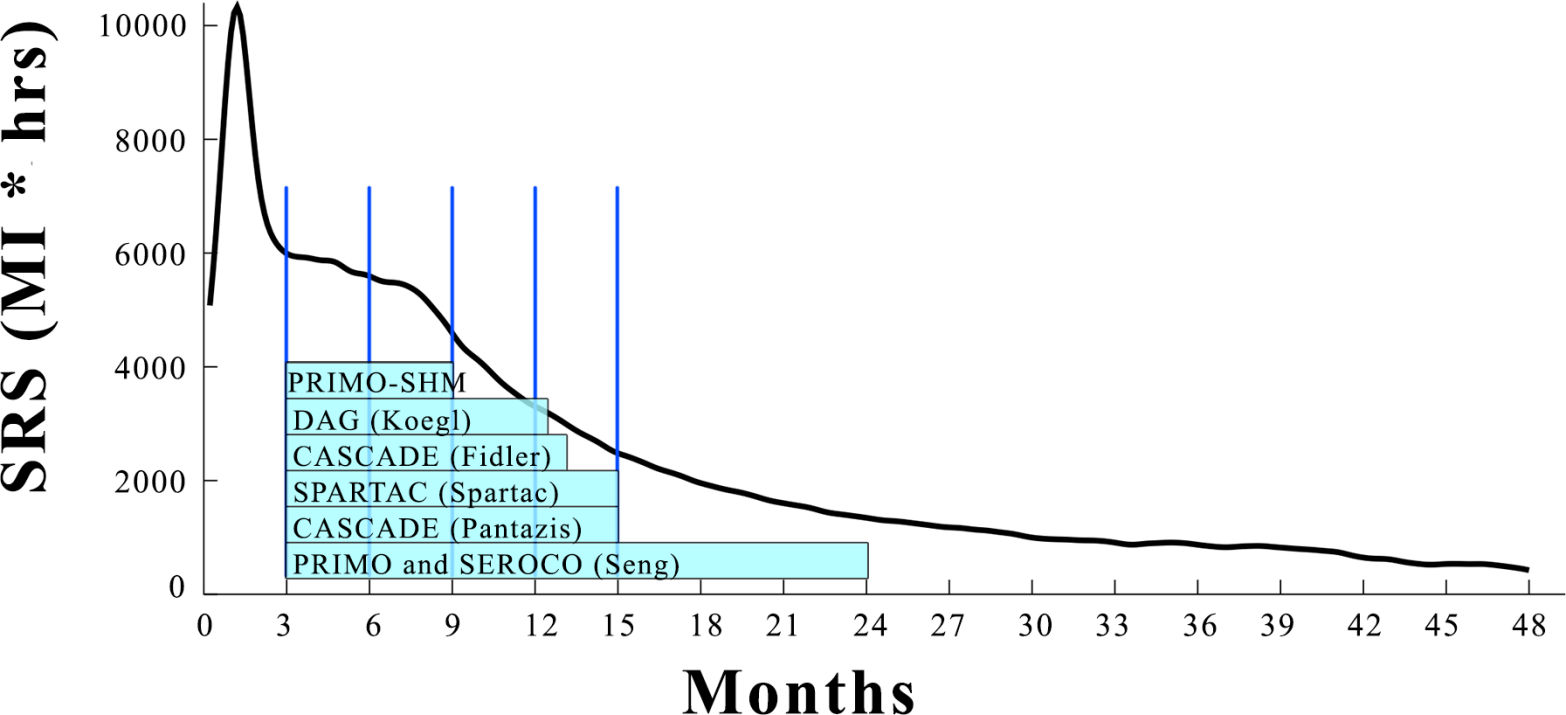
Comparison of the duration of the short course antiretroviral treatments in different clinical trials. We assume randomization in all clinical trial started on average at 3 months after the actual time of infection. The clinical trial that reported the strongest beneficial effects is PRIMO-SHM [18]. SPARTAC trial 48 weeks cART [11], CASCADE [42], Prime-DAG and AC-DAG cohorts [43], and CASCADE [9] reported beneficial effects but not as strong as the ones observed in PRIMO-SHM. PRIMO and SEROCO [13] cohorts reported no beneficial effects at all for the temporary cART schedule tested.

In Fig 7 we compare the duration of short course cART tested in several clinical trials with our predicted SRS curve. All reported trials used patients for whom seroconversion happened in the previous 6 months. We assume that on average this corresponds to 3 months post infection considering the average delay between actual infection and the seroconversion date. The clinical trials that reported beneficial effects of short course cART are those in which treatment was completed within the plateau of our SRS. The beneficial effects diminished as treatment is prolonged in the exponentially declining part and completely lost in PRIMO and SEROCO trials as treatment reached the baseline of the SRS curve.

## Discussion

The PRIMO-SHM clinical study showed that starting a temporary cART within 6 months post infection lowered the viral set point. Beneficial effects were also reported by the SPARTAC group [11] although the extent of such effects were not as strong. However, it is unknown to what extent this effect was due to treatment started during the acute phase or to treatment started after the acute phase. It is known that temporary cART administered during the acute phase has a life long effect, but it is unclear how this effectiveness decreases over time. We predict a phase of long-lasting sustained response during the acute phase followed by a plateau of roughly ten months during which the sustained response of the immune system is more intense than the one relative to the chronic phase. Our results provide additional support to the observed temporary beneficial effect of cART reported in the PRIMO-SHM study and in SPARTAC study. Our simulations show that the driving process is the reduction of the size of HIV reservoir (see Fig 6 inset). Our study presents an explanation to the apparently contradictory reports of different clinical trials and indicates that extending short course cART after the plateau in the response curve does not provide any additional beneficial effect.

It is well known that cART is associated to an increase in the CD4+ cells count at any time during the HIV infection. Less is known about the effects of cART on HIV reservoirs at different stages of the infection. In our analysis the size of HIV reservoirs evaluated by the provirus quantitation is reduced by the use of temporary cART for a period of about 1 year after treatment interruption. The correlation between the survival of patients and the size of the population of infected cells has been observed in *in silico* experiments [21], highlighting the importance of controlling HIV reservoirs to reduce long term mortality. The temporary reduction of the size of HIV reservoirs confirms the previously observed lack of long-term effects of treatment started after the acute infection. The inability of the immune system to identify and eliminate latently infected cells of the HIV reservoirs might be a possible explanation of the slow decrease of Provirus under cART.

In conclusion, our results indicate the existence of a previously unknown phase in HIV dynamics, a plateau phase during which the size of HIV reservoir can still be sustainably affected by treatment. Moreover, our evidence supports the need to detect and treat very early infection when the immunologic benefit is superior as compared to that obtained by treating HIV during both the 10 months window of opportunity and the chronic infection. Taking into account a three phase dynamics for HIV infection (Acute/Plateau/Chronic) might help to define guidelines for treatment interruptions in clinical studies and might improve the predictive power of models for the HIV pandemics since the effects of treating patients during the period of 10 months post infection are not currently taken into account. Finally, the identified "window of opportunity" may be relevant for future research aimed at preserving immune function and containing the viral reservoir as a support to innovative future approaches to a cure for HIV.
